# Integrating genome-wide co-association and gene expression to identify putative regulators and predictors of feed efficiency in pigs

**DOI:** 10.1186/s12711-019-0490-6

**Published:** 2019-09-02

**Authors:** Yuliaxis Ramayo-Caldas, Emilio Mármol-Sánchez, Maria Ballester, Juan Pablo Sánchez, Rayner González-Prendes, Marcel Amills, Raquel Quintanilla

**Affiliations:** 1 Animal Breeding and Genetics Program, Institute for Research and Technology in Food and Agriculture (IRTA), Torre Marimon, 08140 Caldes de Montbui, Spain; 2 Department of Animal Genetics, Centre for Research in Agricultural Genomics (CRAG), CSCIC-IRTA-UAB-UB, Campus de LA Universitat Autònoma de Barcelona, 08193 Bellaterra, Spain; 3 Departament de Ciència Animal i dels Aliments, Universitat Autònoma de Barcelona, 08193 Bellaterra, Spain

## Abstract

**Background:**

Feed efficiency (FE) has a major impact on the economic sustainability of pig production. We used a systems-based approach that integrates single nucleotide polymorphism (SNP) co-association and gene-expression data to identify candidate genes, biological pathways, and potential predictors of FE in a Duroc pig population.

**Results:**

We applied an association weight matrix (AWM) approach to analyse the results from genome-wide association studies (GWAS) for nine FE associated and production traits using 31K SNPs by defining residual feed intake (RFI) as the target phenotype. The resulting co-association network was formed by 829 SNPs. Additive effects of this SNP panel explained 61% of the phenotypic variance of RFI, and the resulting phenotype prediction accuracy estimated by cross-validation was 0.65 (vs. 0.20 using pedigree-based best linear unbiased prediction and 0.12 using the 31K SNPs). Sixty-eight transcription factor (TF) genes were identified in the co-association network; based on the lossless approach, the putative main regulators were *COPS5*, *GTF2H5*, *RUNX1*, *HDAC4*, *ESR1*, *USP16*, *SMARCA2* and *GTF2F2*. Furthermore, gene expression data of the *gluteus medius* muscle was explored through differential expression and multivariate analyses. A list of candidate genes showing functional and/or structural associations with FE was elaborated based on results from both AWM and gene expression analyses, and included the aforementioned TF genes and other ones that have key roles in metabolism, e.g. *ESRRG*, *RXRG*, *PPARGC1A*, *TCF7L2*, *LHX4*, *MAML2*, *NFATC3*, *NFKBIZ*, *TCEA1*, *CDCA7L*, *LZTFL1* or *CBFB*. The most enriched biological pathways in this list were associated with behaviour, immunity, nervous system, and neurotransmitters, including melatonin, glutamate receptor, and gustation pathways. Finally, an expression GWAS allowed identifying 269 SNPs associated with the candidate genes’ expression (eSNPs). Addition of these eSNPs to the AWM panel of 829 SNPs did not improve the accuracy of genomic predictions.

**Conclusions:**

Candidate genes that have a direct or indirect effect on FE-related traits belong to various biological processes that are mainly related to immunity, behaviour, energy metabolism, and the nervous system. The pituitary gland, hypothalamus and thyroid axis, and estrogen signalling play fundamental roles in the regulation of FE in pigs. The 829 selected SNPs explained 61% of the phenotypic variance of RFI, which constitutes a promising perspective for applying genetic selection on FE relying on molecular-based prediction.

## Background

Improving feed efficiency (FE) has become a relevant but challenging focus for pig breeding selection schemes due to its strong influence on the economic sustainability and environmental impact of pig production. FE is a complex phenotype that depends on genetics [1–3], on the health and physiological status of the animals [4], on environmental factors [5, 6], and on the gut microbial composition [7–9]. An additional complexity for genetic improvement of FE is the definition of adequate selection criteria relative to feed intake and other production traits, such as growth, which in turn show a mutual dependency. Besides the conventional feed conversion ratio (FCR), the most widely used measure of FE during the last decade is residual feed intake (RFI), i.e. the deviation of the animal’s feed intake from the amount of feed predicted to be required for maintenance, growth, and back fat deposition [10]. Estimates of the heritability of RFI range from 0.14 to 0.53 [1–3], whereas the reported accuracies of RFI genomic prediction range from 0.40 to 0.53 [11, 12]. Selection for RFI has proven to be a successful strategy for improving FE in pigs [1, 2, 13] but requires recording of individual feed intake, body weight gain, and back fat, which is expensive and time-consuming. Thus, identifying candidate genes and potential regulators of FE that are predictive of the animal’s genetic potential for this phenotype is of paramount interest.

Several studies at both the genomic and transcriptomic levels have been performed to identify candidate single nucleotide polymorphisms (SNPs) associated to FE and to unravel the genetic architecture of this complex trait in pigs [14–16], including genome-wide association studies (GWAS) [14, 17] and whole-genome expression analyses [15, 16, 18]. Among the tissues that are relevant for FE, several studies have focused on the muscle transcriptome [19–21]. Muscle plays a central role in maintaining overall energy balance by controlling the storage of lipids and carbohydrates [22, 23]. However, the results from different studies are not always consistent and most studies ignore gene-by-gene interactions and fail to integrate structural and functional genomics data. Holistic approaches that combine multiple sources of information increase the power to identify candidate genes [24, 25] and provide a more complete picture of the biological processes under investigation. To date, a limited number of studies targeting FE in pigs and other livestock species have implemented integrative approaches, such as system genetics- or gene network-based methods [26–28].

The objective of our study was to use a systems-based approach that integrates information from several FE-related phenotypes, SNP co-associated networks, and gene-expression data to disentangle the molecular mechanisms that underlie FE in pigs. Our aim was also to identify candidate genes and their potential regulators in order to develop a panel of markers that can be used as predictors of an individual’s genetic potential for FE.

## Methods

In this study, we integrated several genome and gene expression analyses that aimed at identifying candidate genes, putative regulators, and predictors of FE in pigs. The different approaches used are described below and outlined in Additional file 1: Figure S1.

### Animals and phenotypes

A population of 350 Duroc barrows from five paternal half-sib families was used in this experiment. Pigs were raised under intensive standard conditions at the Institut de Recerca i Tecnologia Agroalimentàries (IRTA) Experimental Pig Farm in Monells (Girona). They were distributed across four fattening batches in a partially balanced but connected design: one batch included the offspring of all five sires, while the remaining batches contained the offspring of four of the five sires. All animals were subjected to the same management procedures, with ad libitum access to feed, with two standard diets with energy densities of 10.27 MJ/kg until the animals reached 90 kg of live weight (▲150 days of age), and of 9.94 MJ/kg during the last ▼40 days before slaughter. A detailed description of the experimental population and management conditions is in Gallardo et al. [29] and Quintanilla et al. [30]. Animal care and experimental procedures were performed by following national and institutional guidelines for the Good Experimental Practices and were approved by the IRTA Ethical Committee. Pigs were weighed individually at ~ 65 days of age and every 3 weeks during the fattening period, plus on the day of slaughtering (~ 190 days of age). Backfat thickness (BF) was also measured every 3 weeks using PIGLOG 105 ultrasound equipment. Individual feed intake was recorded by electronic feeders located in each pen (IVO^®^-feeding station, Insentec, Marknesse, The Netherlands). The average daily feed intake (ADFI) of each individual during the trial was computed. Two measures of individual FE during fattening were computed: FCR, measured as the simple ratio of ADFI and average daily gain (ADG) (kg/kg), and RFI, computed as the residual of the following model:where $ADFI_{ij}$ is the average daily feed intake of individual $i$ (in batch $j$) during the whole fattening period (from ~ 70 to ~ 190 days of age); $b_{j}$ is the effect of batch level $j$(four batches); $A_{i}$ is the age of individual $i$ at the midpoint of the analysed period (135 days on average but ranging from 121 to 148 days), and $\alpha$ is the corresponding regression coefficient; $MW_{i}$, $ADG_{ij}$ and $BF_{i}$ are, respectively, the metabolic weight (computed as body weight 0.75) at the midpoint of the trial, the ADG during the period, and the BF at the end of the period for individual $i$; $\gamma_{\left( j \right)}$,  and $\delta_{\left( j \right)}$, are the corresponding partial regressions coefficients nested within batch; and $RFI_{ij}$ is the residual feed intake of individual $i$.

Pigs were slaughtered at an approximate age of 190 days (average live weight of 122 kg). After recording live body weight and BF in vivo, pigs were slaughtered according to a commercial protocol. Carcass weight (CW) was registered, the killing out percentage (KO, %) computed, and lean percentage (LEAN) was inferred based on fat and muscle thickness data measured with an AutoFOM ultrasound device. Finally, the percentage of intramuscular fat content (IMF) was determined on a sample of *gluteus medius* (GM) muscle by near infrared transmittance (NIT, Infratec ® 1625, Tecator Hoganas, Sweden), as described in [30, 31].

### Genotype information

Genome-wide SNP genotyping of the 350 Duroc pigs was performed using the porcine SNP60 BeadChip (Illumina, San Diego, CA), which contains 62,163 SNPs. SNPs with a minor allele frequency (MAF) lower than 5%, a rate of missing genotypes higher than 10%, and those that did not conform to Hardy–Weinberg expectations (threshold set at a *p* value of 0.001) were filtered out. We also excluded SNPs that did not map to the porcine reference genome (Sscrofa11.1 assembly) and that were located on the X chromosome. After these filtering steps, we obtained a subset of 30,096 SNPs that were used in the GWAS and in the expression GWAS (eGWAS). Quality control of genotypes and the filtering steps were performed with the GenomeStudio (Illumina) and PLINK [32] programs, respectively.

### Association weight matrix (AWM) and network analysis

The association weight matrix (AWM), which has been applied in previous studies [33–35], allows gene co-association networks with regulatory significance to be generated by combining GWAS results with network inference algorithms. In this study, we used the AWM approach to identify candidate genes and regulators that underlie FE. Nine phenotypes were considered in the analysis: RFI, FCR, ADFI, ADG, CW, IMF, KO, BF and LEAN. Since the most accepted FE measure is RFI, it was set as the key (target) phenotype in the AWM procedure, whereas the other traits were selected based on their association with FE, but also on their relevance in pig production. In a first step, a GWAS was performed for each of the nine aforementioned traits by using the genome-wide complex trait analysis (GCTA) software [36]. The additive effects of each SNP ($k$) on each trait were estimated according to the following model:

$$y_{ij} = b_{j} + \beta age_{i} + u_{i} + s_{ik} a_{k} + e_{ij} ,$$

where $y_{ij}$ corresponds to the phenotype of the $i$th individual in the $j$th batch; $b_{j}$ corresponds to the $j$th batch effect (4 levels); $age_{i}$ is the covariate of age at slaughter of individual $i$, and $\beta$ is the corresponding regression coefficient; $u_{i}$ is the infinitesimal genetic effect of individual $i$, with ${\mathbf{u}}\sim N\left( {0,{\mathbf{G}}\sigma_{u}^{2} } \right)$, where ${\mathbf{G}}$ is the genomic relationship matrix (GRM) calculated using the filtered autosomal SNPs based on the methodology of Yang et al. [18], and $\sigma_{u}^{2}$ is the additive genetic variance; $s_{ik}$ is the genotype (coded as 0,1,2) of individual $i$ for the $k$th SNP, and $a_{k}$ is the allele substitution effect of SNP $k$ on the trait under study; and $e_{ij}$ is the residual term.

Following a previously published procedure [33], in order to build the AWM, we retained the SNPs that were associated (nominal p-value < 0.05) with RFI (target trait) and/or with three or more of the remaining eight phenotypes. Considering both cis-action and the extent of linkage disequilibrium (LD) in pigs, only the SNPs that were located within or less than 10 kb from the nearest annotated gene (Sscrofa11.1 assembly) were retained. Next, we used the z-scores of the estimated allele substitution effects of the SNPs to build the AWM matrix of dimension number of retained SNPs (rows) per number of traits (columns). Hierarchical clustering of traits from allele substitution effects of SNPs was estimated and visualized using the ‘hclust’ R function. The AWM matrix allowed us to explore both correlations between traits based on SNP additive effects (column-wise) and gene-by-gene interactions (row-wise). Gene-by-gene interactions were predicted using the partial correlations and information theory (PCIT) algorithm [37]. The resulting network was used to identify potential regulators by focusing on the transcription factors (TF) within the network. Once the TF were identified, we applied an information lossless approach [34] to the inferred co-association network, which explored the combinations (trios and quartets) of TF that spanned most of the network topology with minimum redundancy.

### Gene expression data

Gene expression profiles in muscle were obtained for three groups of 35 pigs with high, medium, and low lipid metabolism, respectively, which were selected from the 350 pigs in the population by a principal components analysis, as described in Ref. [31]. The objective, here, was to select animals with divergent profiles regarding fat deposition and lipid metabolism, but these animals covered the whole spectrum of the population’s variability regarding RFI and the other analysed traits. GM muscle samples from these 104 pigs were immediately collected after slaughter and snap-frozen in liquid nitrogen until storage at − 80 °C. Total RNA was extracted by using the acid/phenol method [38] implemented in the Ribopure isolation kit (Ambion, Austin, TX). The mRNA expression profile of each sample was characterized by hybridization to the GeneChip Porcine Genome Array (Affymetrix Inc., Santa Clara, CA), which includes 23,998 probes, in two laboratories (66 and 38 samples in each laboratory). Details about RNA isolation and microarray hybridisation procedures are in [31]. Pre-processing, background correction, normalization, and log-transformation of the expression data were performed by computing a robust multi-array average (RMA) per probe [39]. The gene intensity significance level for detecting expressed probes was calculated by using the MAS 5.0 algorithm [39]. Control probes and probes for which the expression level was lower than the detection threshold in 75% of the pigs were discarded for further analyses. The remaining probes were mapped to the *Sus scrofa* genome assembly (Sscrofa11.1) using the Biomart database available at the Ensembl repository (https://www.ensembl.org/biomart/martview/). Expression values for probes that mapped to the same locus were averaged in order to obtain a global estimate of transcript expression at the gene level. Probes that failed to map to a known gene were also removed. The effect of the laboratory where microarrays were assayed on gene expression levels was estimated to be an additive effect, thus, a systematic laboratory effect was included in subsequent analyses of the gene expression data.

### Differential expression and multivariate analyses of expression data

Twenty pigs with extreme RFI were selected for differential expression (DE) analysis: 10 highly feed-efficient (HFE) pigs with low RFI and 10 lowly feed-efficient (LFE) pigs with high RFI. Offspring of four sires were present in both the HFE and LFE groups and all 20 animals had different dams. The DE analysis between HFE and LFE animals was done by following the limma-trend pipeline recommendations [40, 41], fitting a model with batch and laboratory effects, in addition to FE-group. The limma’s empirical Bayes procedure was modified to incorporate a mean–variance trend that models the relationship between variance and gene signal intensity. Fold-change (FC) was computed as the difference between the logarithms of mean expression levels in LFE and HFE pigs, i.e. a positive FC corresponds to higher expression in the LFE group compared to their HFE counterparts. Genes were considered to be DE when |FC| was higher than 1.5 and the q-value lower than 0.05, after adjusting for multiple-testing with the false discovery rate method [42].

The same dataset of the expression level of 7007 genes on 20 extreme pigs for RFI was used in a multivariate framework to perform a sparse partial least squares discriminant analysis (sPLS-DA) [43] in order to identify a subset of genes that discriminated samples according to RFI classification (HFE vs. LFE). In a first step, we determined the classification error rates for the sample group assignation with respect to the number of variables (genes) selected for each component. The classification error rates and optimal number of variables to select for each component are represented in Additional file 1: Figure S2. The classification performance of the final model was assessed in a fivefold cross-validation repeated 500 times, by a function of the maximum distance between overall misclassification error rate and balanced error rate that considers the proportion of incorrectly classified samples weighted by the number of samples per group.

Finally, a regularized canonical correlation analysis (rCCA) was performed using the whole expression dataset for all 104 individuals. The rCCA is an unsupervised multivariate approach to identify subsets of canonical variables that maximize the correlation between two datasets ${\mathbf{X}}$ and ${\mathbf{Y}}$, of sizes (n × p) and (n × q), respectively [43]. In our analysis, ${\mathbf{X}}$ was the matrix of phenotypes for all 104 animals for the traits that were most directly associated with FE (i.e. RFI, FCR, ADG and ADFI) and ${\mathbf{Y}}$ was a matrix with gene expression values for all 104 animals. The shrinkage method was used to tune the regularization parameters λ_1_ and λ_2_, with shrinkage values of λ_1_ = 0.121138 and λ_2_ = 0.128887. Instead of considering all genes that were included in the first canonical component (CC1), we decided to apply a more conservative approach and keep as candidates only the genes for which the correlation between gene expression and FE related traits (RFI, FCR, ADG and ADFI) was higher than 0.29 (median + 2*SD).

### Expression-based genome-wide association studies (eGWAS)

The aim of the eGWAS was to identify SNPs that are associated with the expression of the candidate genes identified in the AWM approach, as well as genes reported by at least two of the three following methods mentioned before: rCCA, sPLS-DA and DE. The association of each SNP with the expression of each gene was estimated by fitting the following model using the GCTA software [36]:

$$y_{ijl} = b_{j} + l_{l} + u_{i} + s_{ik} a_{k} + e_{ijl} ,$$

where $y_{ijl}$ corresponds to the gene expression of individual $i$ raised in batch $j$ and processed in laboratory $l$; $b_{j}$, $u_{i}$, $a$ and $e_{ijl}$ are as defined in the previous GWAS model; and $l_{l}$ is the fixed effect of the laboratory where the microarray was assayed (2 levels). After multiple testing adjustment, the cut-off for a significant association at the whole-genome level was established at a q-value ≤ 0.05.

### Gene functional classification and canonical pathway analyses

Functional classification and pathway analyses of the list of candidate genes were carried out using the Ingenuity Pathways Analysis software (IPA; Ingenuity Systems, http://www.ingenuity.com). Significance levels for enrichment of each canonical pathway in the list of candidate genes were calculated using Fisher’s exact test and the resulting p-values were corrected for multiple-test using the Benjamini and Hochberg algorithm [42]; the cut-off for considering an enrichment as significant was established at a corrected p-value < 0.05.

### Proportion of phenotypic variance explained by the SNPs and their prediction accuracy

The proportion of phenotypic variance of RFI that was explained by the SNPs identified in the previous analyses was estimated by a Bayesian Gibbs sampling approach, using the gibbs2f90 program included in the Blupf90 package [36, 44]. We performed three estimations of genomic variance by considering different subsets of SNPs: (1) SNPs that were selected by the AWM procedures (829 SNPs); (2) the former AWM-SNPs plus the eSNPs identified by the eGWAS (1078 SNPs); and (3) all SNPs that passed quality controls (~ 31K SNPs). The single-step method [45] implemented in Blupf90 was used. Subsequently, we also performed a pedigree-based estimation of RFI heritability, in order to define a baseline for comparing the proportion of variance explained by the SNPs.

All estimates of variance components were obtained with the following animal model:

$$RFI_{ij} = b_{j} + \beta age_{i} + u_{i} + e_{ij} ,$$

where all terms are defined as previously described for the GWAS. For the Bayesian implementation, the additive genetic effect $u_{i}$ was assumed to follow a normal distribution with a mean of zero and different (co)variance matrices depending on the analysis: (1) ${\mathbf{K}}\sigma_{u}^{2}$ for genomic-based estimations, where ${\mathbf{K}}$ is the genomic relationship matrix computed for the different SNP subsets, as described in [46]; and (2) ${\mathbf{A}}\sigma_{u}^{2}$ for pedigree-based estimation, where ${\mathbf{A}}$ is the pedigree-based numerator relationship matrix. A *prior* uniform distribution was assumed for batch and age effects. The Gibbs sampler algorithm was run for 100,000 iterations with a burn-in of 10,000 rounds, and then saving one out each 10 samples.

The accuracy for predicting RFI phenotype based on the different sources of genetic (pedigree) and genomic (all SNPs, and AWM or AWM-eGWAS subsets of SNPs) information was assessed by cross-validation. The cross-validation scheme comprised 20 random replicates. In each replicate, the whole dataset was split randomly into training and validation datasets that contained approximately 88 and 12% of records, respectively. The training dataset was used to predict the genetic additive effects of SNPs by solving the mixed model equations (blupf90) with variance components estimates that were obtained with the complete dataset. Subsequently, phenotypes in the validation dataset were predicted from model solutions obtained in the training set and the prediction accuracy was defined as the correlation coefficient between predicted and observed records in the validation dataset. The accuracy of each model/SNP subset was computed by averaging correlations across replicates.

## Results

### Phenotypes analysed

In our study, we focused on the genetic regulation of FE, but we considered nine traits that are directly or indirectly associated with FE, such as growth rate, feed intake, carcass traits, and fat deposition. The analysed pigs belonged to a commercial Duroc line that is used to produce highly cured products and characterised by its high IMF depot. Summary statistics of the phenotypes considered for the analysed population (350 pigs) are in Table 1. Due to being a residual term, RFI has a mean of zero, whereas the mean for FCR indicated that, on average, the analysed Duroc pigs consumed 3.16 kg of feed for 1 kg of growth. The values for the remaining phenotypes are consistent with the general characteristics of this Duroc line: carcasses weighing ~ 95 kg with a KO percentage of ~ 75%; high subcutaneous fat deposition (mean BF of 24 mm) and intramuscular compartments (mean IMF in GM reaches values > 5%), and a low lean percentage in the carcass of 40.8% compared with other breeds [47]. It should be noted that age and weight at slaughter were substantially greater than the values normally reached in commercial production conditions. Phenotypic means in the two groups selected for extreme RFI (HFE and LFE) are also in Table 1. The two groups diverged significantly for all FE phenotypes, particularly in the classification criterion RFI (mean values of − 0.30 vs. 0.26 kg/d in the HFE vs. LFE groups) but also in FCR (mean values of 2.72 vs. 3.30 for the HFE vs. LFE pigs, p-value < 10^−5^). These two groups of animals also differed in feed consumption and production traits, with the HFE animals displaying lower feed intake (0.70 kg less per day), smaller weight at slaughter (carcasses 9 kg lighter), higher lean content (42.6 vs. 37.0%) and lower IMF (4.8 vs. 7.7%) than the LFE pigs.

**Table 1:** Mean (standard deviation) of the analysed phenotypes in the population of 350 individuals and in the two extreme groups (10 individuals each) for RFI, denoted as HFE and LFE (high and low feed efficiency groups, respectively), and the significance (p-value) of differences between HFE and LFE groups

Phenotype	Mean (SD) N = 350	Mean HFE (N = 10)	Mean LFE (N = 10)	p-value^a^
RFI (kg/day)	0.00 (0.17)	− 0.30	0.26	< 10^−11^
FCR (kg feed/kg gain)	3.16 (0.31)	2.72	3.30	< 10^−5^
ADFI (kg/day)	2.81 (0.37)	2.31	3.06	< 10^−4^
ADG (kg/day)	0.89 (0.11)	0.85	0.93	n.s.
CW (kg)	94.8 (10.6)	89.0	98.6	< 10^−1^
KO (%)	74.8 (2.4)	73.4	76.6	< 10^−1^
BF (mm)	24.0 (4.9)	22.4	24.5	n.s.
Lean (%)	40.8 (4.5)	42.6	36.8	< 10^−1^
IMF (%)	5.23 (2.06)	4.83	7.68	< 10^−2^

RFI, residual feed intake; FCR, feed conversion ratio; ADFI, average daily feed intake; ADG, average daily gain; BF, back fat thickness; CW, carcass weight; KO, killing out percentage; LEAN, lean percentage; IMF, intramuscular fat content; n.s., non-significant differences between groups

^a^Student t test of LS means for group effect

Correlation coefficients between the analysed phenotypes (r_P_) are in Table 2. High but less than 1 phenotypic correlations (r_P_ = 0.68) were observed between the two FE indicators RFI and FCR. Both these traits were significantly correlated with feed intake (r_P_ = 0.46 and 0.43 for RFI and FCR, respectively). Conversely, only FCR was associated with ADG and BF, because RFI was the residual term from a regression function that encompassed ADG and BF and was, therefore, independent from these two phenotypes. The strongest phenotypic relationships were observed between feed intake (ADFI), growth (ADG), and CW (r_P_ ranging from 0.71 to 0.81). Subcutaneous fat deposition (BF) showed a moderate to high correlation with all traits, except with RFI and KO, whereas IMF had a low to moderate, but always significant, correlation with all analysed traits, including RFI. LEAN displayed negative associations with fat deposition traits (BF and IMF), but also with ADG, ADFI and RFI. Finally, the phenotypic correlations of KO with the other traits were negligible, except with CW.

**Table 2:** Estimates of phenotypic (above the diagonal) and AWM-genomic (below the diagonal) correlations between the analysed phenotypes and their significance

	RFI	FCR	ADFI	ADG	CW	KO	BF	Lean	IMF
RFI		0.677^***^	0.461^***^	− 0.004 ^ns^	− 0.001 ^ns^	0.055 ^ns^	0.025 ^ns^	− 0.079^*^	0.162^**^
FCR	0.763^***^		0.425^***^	− 0.276^***^	− 0.075 ^ns^	0.025 ^ns^	0.191^**^	− 0.050 ^ns^	0.138^**^
ADFI	0.435^***^	0.177^***^		0.738^***^	0.709^***^	0.087 ^ns^	0.687^***^	− 0.231^***^	0.302^***^
ADG	0.011 ^ns^	− 0.261^**^	0.771^**^		0.811^***^	0.044 ^ns^	0.569^***^	− 0.191^**^	0.205^***^
CW	− 0.061^+^	− 0.282^***^	0.720^***^	0.927^***^		0.338^**^	0.600^***^	− 0.141^+^	0.188^**^
KO	− 0.204^***^	0.422^***^	0.039 ^ns^	0.219^***^	0.422^**^		0.044 ^ns^	0.039 ^ns^	0.029^+^
BF	0.015 ^ns^	− 0.038 ^ns^	0.618^***^	0.740^***^	0.675^***^	0.060^+^		− 0.279^***^	0.352^***^
Lean	− 0.304^***^	− 0.241^***^	− 0.415^***^	− 0.295^***^	− 0.172^**^	0.194^**^	− 0.462^**^		− 0.182^***^
IMF	0.242^***^	0.162^***^	0.481^***^	0.402^***^	0.325^***^	− 0.014^+^	0.607^***^	− 0.451^***^	

RFI, residual feed intake; FCR, feed conversion ratio; ADFI, average daily feed intake; ADG, average daily gain; BF, back fat thickness; CW, carcass weight; KO, killing out percentage; LEAN, lean percentage; IMF, intramuscular fat content; *ns* non-significant

*** p < 0.0001; **p < 0.01; *p < 0.05; + p < 0.1

### Association matrix, gene co-association network, and potential regulators for FE

The GWAS results served as the basis for the AWM approach. After the SNP selection process, 829 SNPs were retained to build the AWM co-association matrix with RFI plus the eight other analysed phenotypes. Consistently with the AWM procedure, most selected SNPs (620 out of 829) were associated with the key phenotype RFI and with on average two other traits, while the remaining 209 SNPs were associated with at least three of the other traits but not with RFI. Annotation of the 829 SNPs that were selected in the AWM procedure identified 879 genes, since several SNPs were annotated to more than one gene. The list of selected SNPs and the corresponding annotated genes is in Additional file 2: Table S1. Among the genes that were associated with several of the analysed traits, we would like to mention those related to the nervous system, such as *sidekick cell adhesion molecule 1* (*SDK1*), *neuronal pentraxin 1* (*NPTX1*), *neuronal guanine nucleotide exchange factor* (*NGEF*), and *catenin delta 2* (*CTNND2*). We also identified genes that are linked to the immune system such as *tyrosine*-*protein kinase JAK1* (*JAK1*) and *DnaJ heat shock protein family (Hsp40) member C6* (*DNAJC6*), which are among the genes that were associated with a large number of traits.

**Fig. 1:**
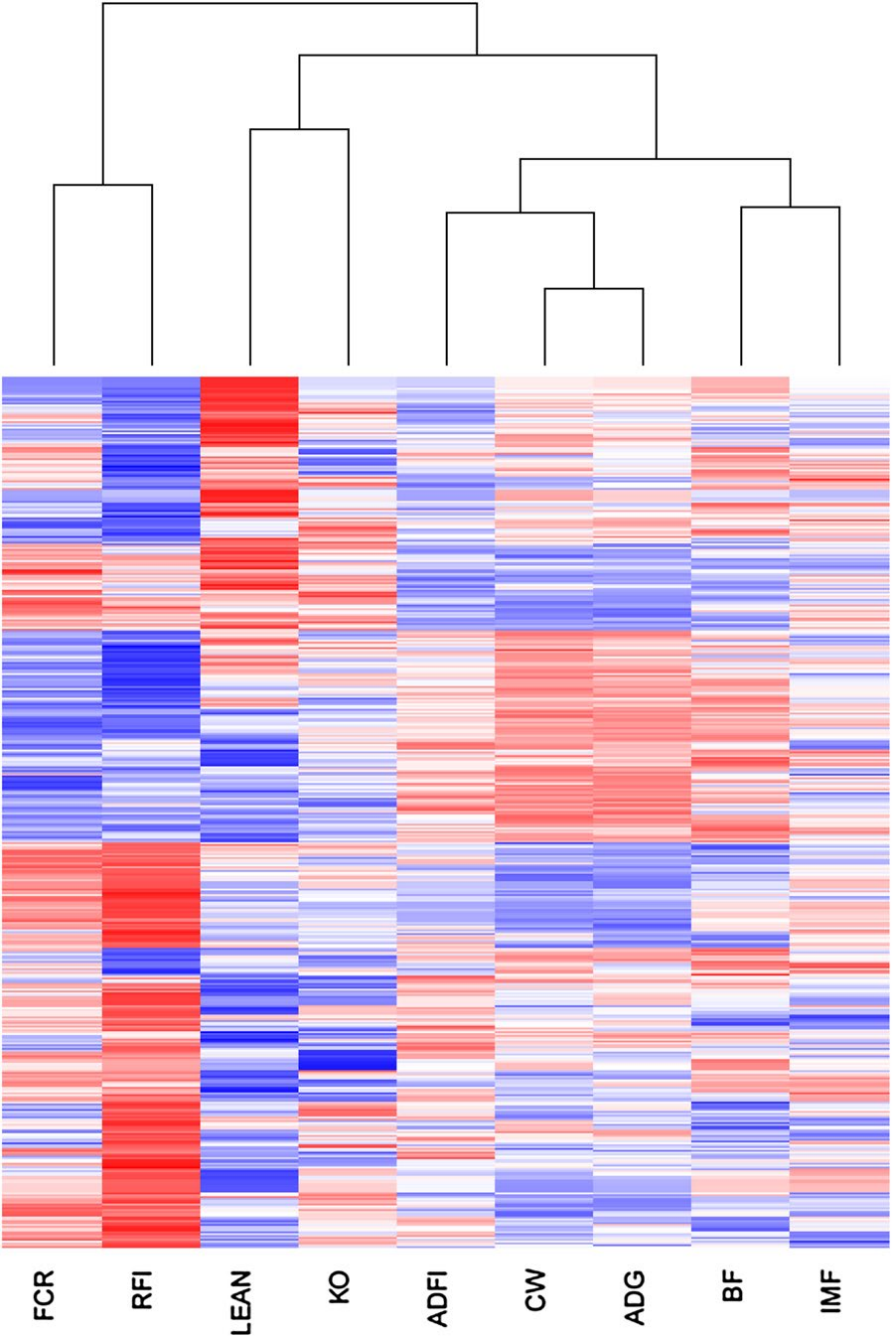
Hierarchical tree cluster of the nine analysed phenotypes obtained from the standardized additive effects of 829 SNPs identified by the AWM procedure. RFI, residual feed intake; FCR, feed conversion ratio; ADFI, average daily feed intake; ADG, average daily gain; BF, back fat thickness; CW, carcass weight; KO%, killing out percentage; LEAN, lean percentage; IMF, intramuscular fat content

Using the AWM columns, correlations between traits were also computed based on the estimates of the standardized allele substitution effects of the set of 829 SNPs across traits. Sample size and experimental design did not allow us to properly estimate genetic correlations between traits; using pedigree information only, the uncertainty regions for these parameters covered the whole parametric space. Therefore, phenotypic correlations (Table 2) were used as a basis to compare the relationships between traits based on SNP effects. The correlation coefficients obtained from AWM were in general consistent with but larger than the estimated phenotypic correlations between traits (Table 2). For instance, the AWM-derived correlation coefficient between RFI and FCR increased to 0.76 (vs. r_P_ = 0.68), and that between BF and IMF was 60% higher than the corresponding phenotypic correlation (r_AWM_ = 0.61 vs. r_P_ = 0.39). In contrast, the correlation between ADG and ADFI captured by AWM was only slightly higher than that the estimated phenotypic correlation (r_AWM_ = 0.77 vs. r_P_ = 0.74). The map of associations between phenotypes based on the AWM additive effects of selected SNPs was not identical to the observed phenotypic relationships between traits, and this result is reflected by the hierarchical tree cluster in Fig. 1, in which traits that are mainly associated with FE, i.e. RFI and FCR, cluster together. This FE block was associated with a second block that included the remaining traits distributed in two groups: Group 1 included growth, feed intake, and fat deposition, which was further subdivided in two blocks: ADFI + ADG + CW and BF + IMF; and Group 2 included LEAN and KO, which clustered together in spite of their negligible phenotypic correlation.

The network derived from the co-association analysis obtained by PCIT gathered 829 nodes, which corresponded to the formerly mentioned 829 SNPs and that were connected by 57,718 significant edges that represented the significant interactions occurring between them. Sixty-eight SNPs mapped to genes classified as TF (indicated as TF in Additional file 2: Table S1) and were thus candidates as potential regulators of the network. As expected, TF genes were the most interacting genes in the network, i.e. the hub in the co-association network topology. Among these genes, *ubiquitin carboxyl*-*terminal hydrolase 16* (*USP16*), *runt*-*related transcription factor 1* (*RUNX1*), and *SWI/SNF*-*related matrix*-*associated actin*-*dependent regulator of chromatin A2* (*SMARCA2*) accumulated the largest number of interactions within the network (more than 220 interactions, each). Other TF genes from the RUNX and FOX (*forkhead box protein*) families, jointly with the *bromodomain PHD finger transcription factor* (*BPTF*), *Wilms tumor 1* (*WT1*), *general transcription factor IIF subunit 2* (*GTF2F2*) or *COP9 signalosome subunit 5* (*COPS5*) genes, can also be included in the list of top TF based on their number of interactions, since they all showed a number of connections larger than average (139).

The information lossless approach allowed the identification of the combinations of TF that spanned most of the network topology with minimum redundancy. These combinations (trios or quartets) of TF are in Table 3. The top trios of regulators spanned a network that gathered between 519 and 521 nodes (out of 829). When a combination of four TF was considered, the regulated network expanded from 603 to 613 nodes. Both types of combinations, trios and quartets, included at least one of the top TF mentioned above (*USP16*, *RUNX1*, *SMARCA2* or *COPS5*; and in quartets also *GTF2F2* or *WT1*), combined with other TF that had a smaller number of but less redundant interactions, such as *general transcription factor IIH subunit 5* (*GTF2H5*), *histone deacetylase 4* (*HDAC4*) or *estrogen receptor 1* (*ESR1*), which was present in three out of six top combinations (Table 3). The co-association network that spanned the maximum number of nodes (613) was linked to the combination of *COPS5*, *ESR1*, *GTF2F2* and *USP16* TF. Other relevant TF included in the network (although not taking part in the top combinations or regulators), that had a large number of interactions (> 100) and that displayed associations with FE were the *LIM homeobox protein 4* (*LHX4*), *mastermind*-*like protein 2* (*MAML2*), and *transcription factor 7 like 2* (*TCF7L2)* genes.

**Table 3:** Combination of regulators (trios and quartets) in the list of 829 SNPs identified by the AWM procedure that spanned most of the network topology obtained by PCIT with minimum redundancy

Combinations	Regulators	Number of target genes
Trios	COPS5-GTF2H5-RUNX1	521
	COPS5-HDAC4-RUNX1	520
	ESR1-SMARCA2-USP16	519
Quartets	COPS5- ESR1-GTF2F2- USP16	613
	COPS5- ESR1-RUNX1- WT1	603
	COPS5- GTF2F2- HDAC4- USP16	603

### RFI prediction based on the SNPs identified with AWM

We evaluated the usefulness of the set of SNPs identified in the AWM procedure to predict RFI phenotype. Following a single-step animal model, the additive effects of the 829 SNPs yielded by the AWM procedure explained about 61% of the phenotypic variance of RFI (Table 4); please note the low error estimated for this parameter. Noteworthy, when all available (31K) SNPs were used for variance component estimation, only 20% of the RFI variance was captured by the additive genetic effects of SNPs. Finally, the pedigree-based estimation yielded a heritability of 0.51 for RFI (Table 4). Although their estimated errors are remarkably large, these latter figures allow us to define a baseline to assess the relevance of the proportion of RFI variance explained by the SNPs identified by AWM.

**Table 4:** Proportion of variance in residual feed intake explained by additive genetic effects (heritability) and correlations between observed and predicted records (prediction accuracy) based on either pedigree or genomic data using three sets of SNPs

	Pedigree	All SNPs	AWM SNPs	AWM + eGWAS SNPs
Heritability^a^	0.51 ± 0.21	0.20 ± 0.11	0.61 ± 0.06	0.66 ± 0.06
Prediction accuracy^b^	0.20 (0.13)	0.12 (0.11)	0.65 (0.15)	0.60 (0.09)

All SNPs, the complete 31K SNP dataset; AWM SNPs, SNPs identified by the association weight matrix (AWM) approach (829 SNPs); AWM + eGWAS SNPs, SNPs identified by AWM and eGWAS (1078 SNPs)

^a^Marginal posterior mean ± marginal posterior standard deviation

^b^Average (standard deviation) across 20 replicates of cross-validation

The expected accuracy for predicting RFI using the SNP panel selected by AWM was assessed by cross-validation. The correlation between actual and predicted RFI using the effects of the 829 SNPs was 0.65 (Table 4). This value is far from that obtained when RFI was predicted by considering all 31K SNP genotypes or when using only pedigree information in a best linear unbiased prediction (BLUP) procedure (prediction accuracies of 0.12 and 0.20, respectively). Finally it is worth mentioning that the mean prediction accuracy obtained with the 620 (out of 829) SNPs that were directly associated to RFI was equal to 0.61, a value that was slightly but not significantly lower than that obtained when all 829 SNPs identified by AWM were considered (Table 4).

### Gene expression and feed efficiency (DE and multivariate analyses)

**Fig. 2:**
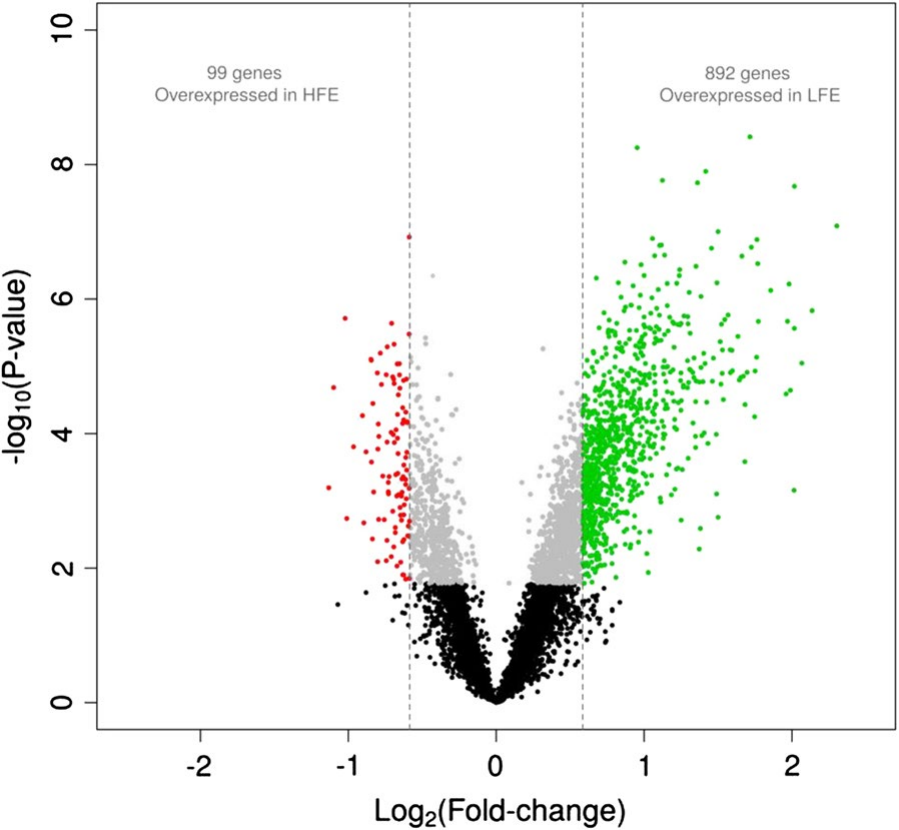
Volcano plot of differential gene expression between the two groups of animals with high and low feed efficiency (HFE and LFE, respectively)

Two groups of animals selected for extreme RFI (HFE and LFE, 10 individuals for each) were used in the DE analysis of muscle. After quality control, this analysis was performed with the expression levels of 7007 genes that were expressed in the GM muscle. A total of 991 were differentially expressed between the HFE and LFE groups (|FC| > 1.5; q-value < 0.05), of which 892 genes had higher expression in the LFE samples (Fig. 2 and Additional file 3: Table S2). The *estrogen related receptor gamma* (*ESRRG*) gene showed the largest differences in expression between the two groups of extreme FE (FC = 4.94; q-value = 8.2*10^−5^). Regulator genes that are involved in energy metabolism and IMF, such as *PPARGC1A,* were also upregulated in the LFE group, as well as the heat shock protein *DNAJC2* gene, which drives the cellular response to heat stress. In contrast, nuclear receptor genes involved in myogenesis such as *retinoid X receptor gamma* (*RXRG*) were upregulated in the more efficient group (HFE). In general, we observed that the genes that were upregulated in LFE pigs belonged to pathways related to protein ubiquitination, valine and isoleucine degradation, IL-6 and IL-8 signalling, glucocorticoid receptor, and estrogen receptor signalling.

**Fig. 3:**
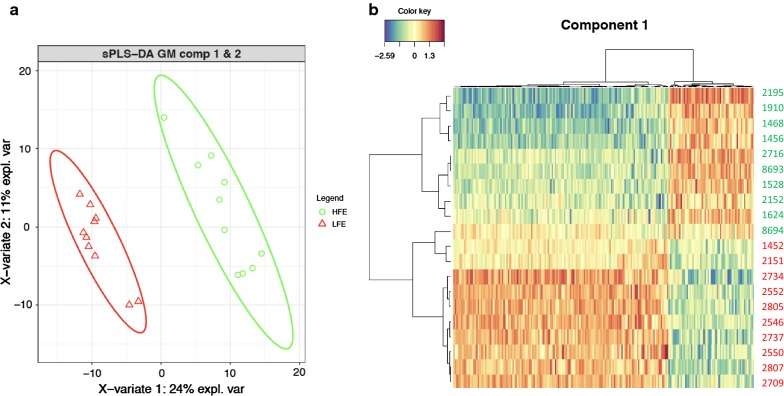
**a** Representation of the samples belonging to animals with high (green) and low (red) feed efficiency according to the two first components of gene expression levels obtained with the discriminant analysis performed by sPLS-DA. **b** Clustering of samples obtained with the 200 genes included in the first component

Regarding the multivariate analyses that were performed with sPLS-DA, the first principal component (PC1) combined the expression pattern of 200 genes, which explained 24% of the total variance in gene expression and allowed a clear discrimination between the two extreme feed efficiency groups (Fig. 3). Supporting this accurate classification, a low balanced error rate ranging from 0.13 (PC1) to 0.08 (PC2) was observed (see Additional file 1: Figure S3). The list of 200 genes included in the PC1 and their corresponding contribution to PC1 is in Additional file 4: Table S3. Importantly, 17 out of the 18 most discriminating genes (*LAMC2*, *RF00278*, *MFSD1*, *ULK2*, *HP1BP3*, *ZNF276*, *BBS4*, *RBBP6*, *PER3*, *PRKAA2*, *TCEA3*, *NFATC3*, *MTUS1*, *SNTB1*, *EIF3F*, *SLC16A5*, and *UHRF1BP1)* were also identified in the DE and/or in the rCCA analyses described below.

**Fig. 4:**
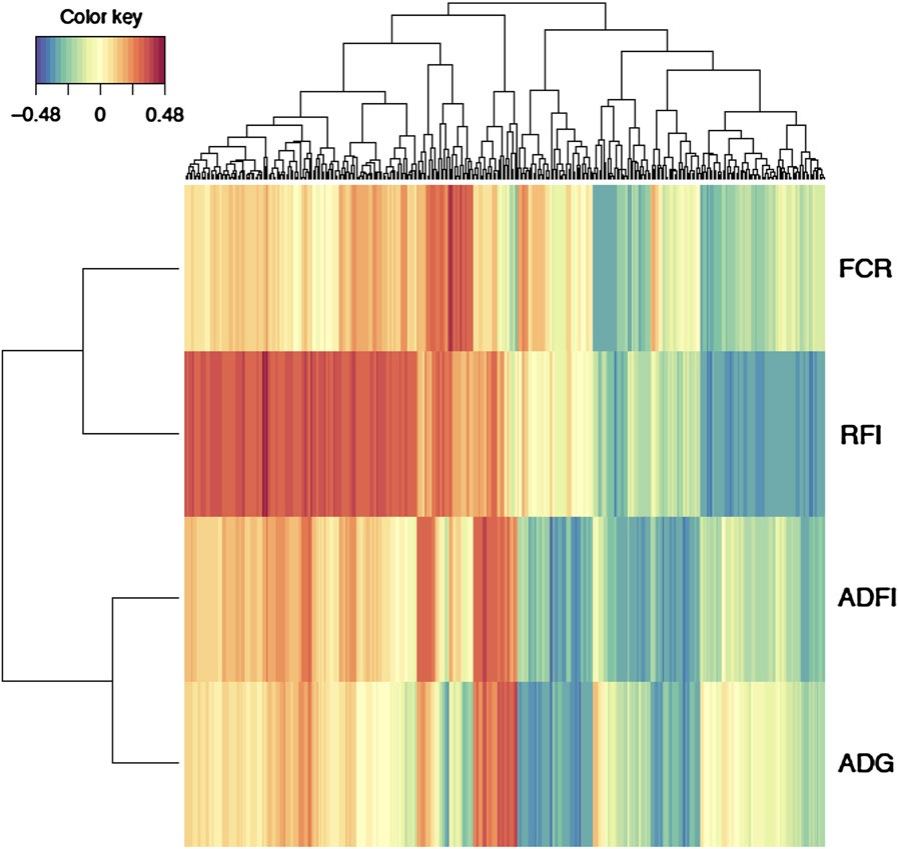
Clustering of RFI, FCR, ADG and ADFI obtained with the 350 genes identified by rCCA

The rCCA procedure allowed us to explore the gene expression and phenotype joint (co)variation by using the whole expression dataset (104 individuals). This analysis yielded 350 genes (see Additional file 5: Table S4) that were included in the first canonical component (CC1), which, in turn, displayed correlations higher than 0.29 with the FE traits. Among these genes, it is worth highlighting the *ESRRG* gene, which was previously shown to have the highest DE between groups of extreme FE. Other relevant genes identified within CC1 were *ALDH1A2*, *NSMAF*, *ARMC6*, *PI15*, *CD163*, *C4BPA*, *PLCXD2*, *MYOD1*, *PIK3R1*, *NNAT*, *ALDH18A1*, *ARHGAP29*, *TMEM158*, *ART3*, *SYBU*, *TMEM98*, and *CCDC71.* Genes included in the CC1 were also used to establish a correlation network between gene expression and phenotype variation for the four traits that were most associated with FE: RFI, FCR, ADG and ADFI. We found that most of the genes from CC1 were correlated with RFI, and that the muscle expression pattern for these genes allowed the clustering of RFI and FCR (Fig. 4), as was the case when co-associated SNPs were used.

**Fig. 5:**
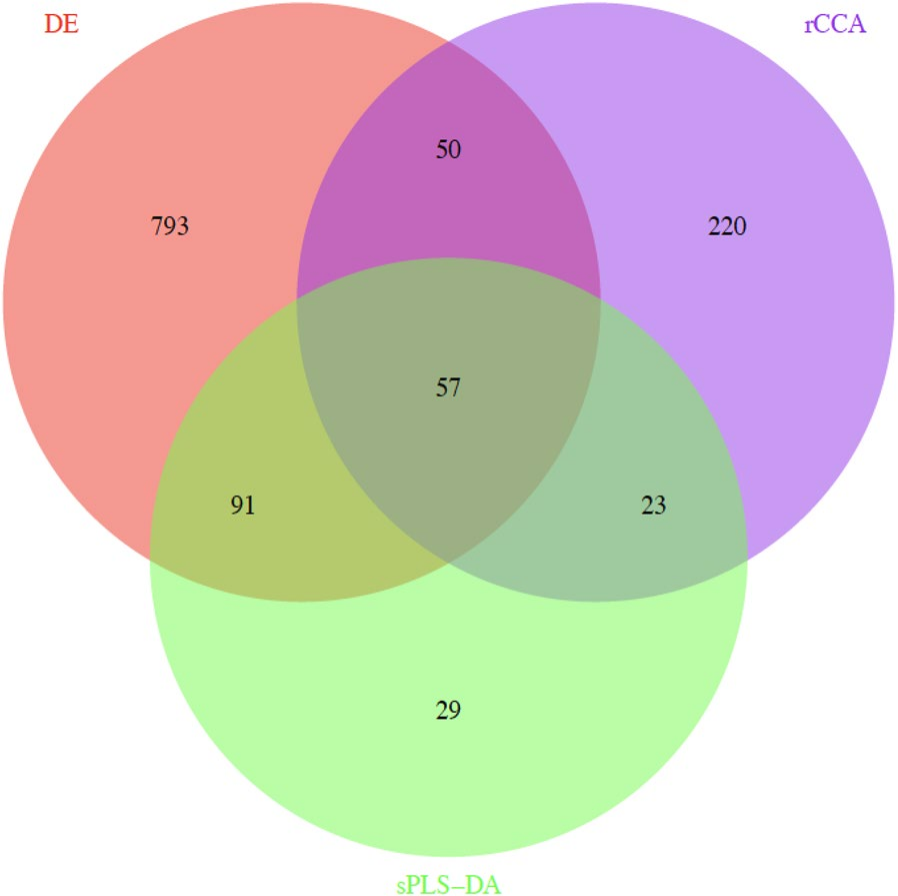
Overlapping genes from the three approaches used for gene expression analysis

**Fig. 6:**
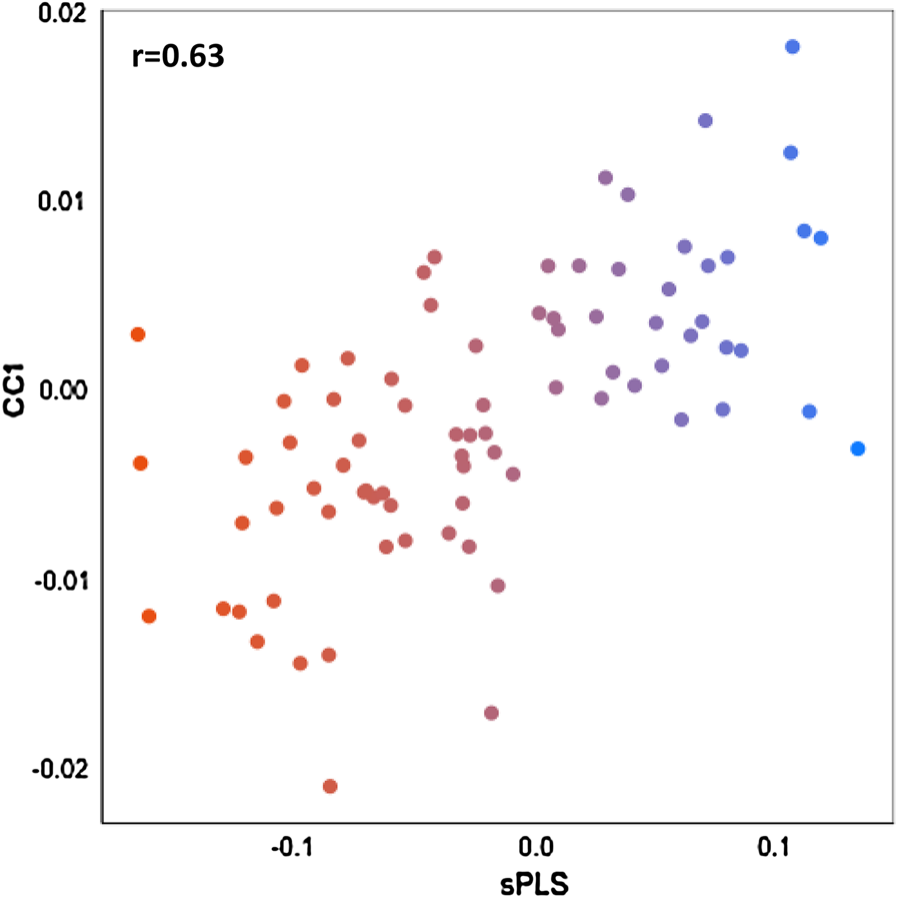
Correlation between the loading factors for the gene expression of the common genes identified with the rCCA and sPLS-DA procedure

In summary, 57 common genes were reported by all three approaches, i.e. DE, sPLS-DA, and rCCA, and this number increased to 221 when genes detected by at least two approaches were considered (Fig. 5). Among the genes for which expression was associated with FE indicator traits (RFI and FCR) and with the other production traits, several TF genes were identified as potential regulators of FE, including *ESRRG*, *ZNF473*, *NFATC3*, *RXRG*, *PPARGC1A*, *NFKBIZ*, *TCEA1*, *CDCA7L*, *ZFP64*, *LZTFL1*, *RBL2*, and *CBFB*. Finally, it is worth mentioning the concordance between the results obtained with different approaches in gene expression analyses. This way, a strong and positive correlation (r = 0.64) was found between the loadings of the 57 common genes in the first principal component (PC1) and the first canonical component (CC1) that were obtained with the sPLS-DA and rCCA procedures, respectively (Fig. 6). Similarly, genes that were up-regulated in the LFE group showed negative loadings in PC1 and were positively correlated with RFI and FCR, whereas genes with a positive weight on PC1 were up-regulated in the HFE group and negatively correlated with RFI and FCR.

### Candidate genes associated to FE and functional classification

**Fig. 7:**
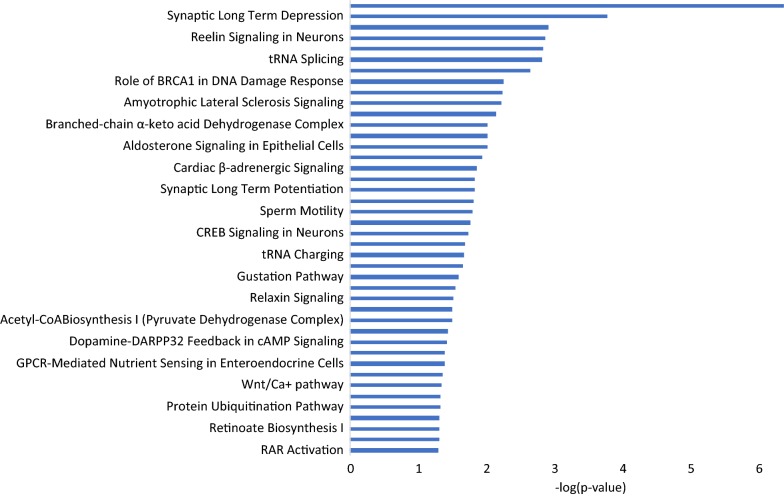
Biological pathways that are over-represented in the list of candidate genes for feed efficiency. The x-axis represents the −log(p-value)

Our results obtained in the structural and functional genomic analyses were combined to compile a list of candidate genes, which included the 879 genes that were present in the co-association network built by AWM and the 221 genes that were identified in at least two of the three approaches used for gene expression analyses. Functional annotation (Fig. 7 and Additional file 6: Table S5) showed that these genes belong to a wide variety of biological processes related to behaviour, immunity, nervous system signalling, and neurotransmitters.

### Identification of eSNPs (eGWAS) for the candidate genes

An eGWAS was performed to identify SNPs that were associated with expression (eSNPs) of candidate genes for FE. We hypothesized that SNPs that regulate the expression of genes that are associated with FE may contribute to improve the molecular-based prediction of RFI. Thus, the final goal was to evaluate the potential increase in predictive ability of RFI that was derived from adding these polymorphisms to the panel of selected SNPs identified by AWM. The eGWAS was performed on 497 genes selected from the aforementioned list of candidate genes by taking into account their mRNA levels in muscle (only 290 of the genes identified by AWM were expressed in GM).

The eGWAS showed significant associations at the genome-wide level (q-value < 0.05) between 269 SNPs (eSNPs) and the expression of 16 of the 497 genes (Table 5 and Additional file 7: Table S6). These 269 eSNPs were distributed across 30 intervals (Table 5) that were located on nine chromosomes (SSC for *Sus scrofa* chromosome, SSC1, SSC2, SSC6, SSC7, SSC8, SSC9, SSC12, SSC14, and SSC17). The largest number of associations was found for the ENSSSCG00000024596 and ENSSSCG00000032907 genes, which included 13.6% (39) and 11.1% (32) of the eSNPs, respectively. In addition, we found that 40.5% of these SNPs (117 eSNPs) regulated gene expression in cis- (i.e. the genome position of eSNP and the target gene map differed by ± 1 Mb) and the remaining 172 eSNPs were associated with the expression level of genes located either on a distant genome region (distance > 1 Mb) or on another chromosome.

**Table 5:** Genome-wide eQTL for 497 candidate genes expressed in the *gluteus medius* (GM) muscle of Duroc pigs

Ensembl ID	SSC	N	SNP	Region (Mb)	q-value	*B*	δ ± SE	A1	MAF
ENSSSCG00000004064	1	27	rs81289665	5.69–10.57	4.34E−03	1.30E−02	− 0.45 ± 0.09	G	0.42
ENSSSCG00000004071	1	6	rs81278169	12.26–13.05	8.75E−04	8.75E−04	0.51 ± 0.09	A	0.40
ENSSSCG00000014432	2	1	rs80929229	149.45–149.45	3.71E−03	2.97E−02	− 0.47 ± 0.10	A	0.10
ENSSSCG00000014432	2	9	rs81290831	150.27–151.35	2.72E−03	1.91E−02	− 0.46 ± 0.09	A	0.11
ENSSSCG00000005976	4	6	rs80965603	14.33–15.13	1.55E−02	3.10E−02	0.24 ± 0.05	C	0.28
ENSSSCG00000005976	4	1	rs81261174	29.68–29.68	4.40E−02	3.08E−01	0.23 ± 0.05	C	0.29
ENSSSCG00000032907	4	3	rs80955389	89.79–89.95	4.01E−02	1.00E+00	− 0.24 ± 0.06	C	0.48
ENSSSCG00000032907	4	32	rs80977488	90.00–99.43	4.25E−04	4.25E−04	0.35 ± 0.06	G	0.36
ENSSSCG00000032907	4	4	rs80889119	103.32–103.47	4.01E−02	1.00E+00	0.31 ± 0.08	G	0.45
ENSSSCG00000033497	6	4	rs81388022	68.99–69.13	1.85E−02	7.41E−02	0.34 ± 0.07	C	0.23
ENSSSCG00000027659	6	25	rs81389074	80.81–82.56	8.02E−04	1.36E−02	− 0.30 ± 0.06	A	0.15
ENSSSCG00000038994	6	19	rs81389074	80.81–82.15	6.32E−03	1.14E−01	0.85 ± 0.18	A	0.15
ENSSSCG00000027659	6	2	rs81309503	111.39–111.40	3.98E−02	1.00E+00	− 0.26 ± 0.06	G	0.15
ENSSSCG00000025087	6	2	rs81393449	156.12–156.13	4.27E−02	8.55E−02	− 0.42 ± 0.09	A	0.09
ENSSSCG00000003844	6	3	rs81345502	157.59–158.65	1.13E−06	1.13E−06	0.30 ± 0.05	G	0.43
ENSSSCG00000003844	6	1	rs81333128	162.74–162.74	2.37E−02	7.10E−02	0.21 ± 0.04	A	0.34
ENSSSCG00000001455	7	12	rs339209635	23.40–26.06	9.64E−03	1.98E−02	0.60 ± 0.12	A	0.49
ENSSSCG00000024596	8	1	rs81237638	37.96–37.96	1.60E−02	6.55E−01	− 0.33 ± 0.08	G	0.33
ENSSSCG00000024596	8	22	rs81402024	83.19–89.67	1.58E−02	1.23E−01	− 0.37 ± 0.08	C	0.37
ENSSSCG00000024596	8	39	rs81402439	90.31–99.95	1.58E−02	2.99E−01	0.34 ± 0.08	G	0.33
ENSSSCG00000024596	8	11	rs81310686	100.02–101.17	1.58E−02	4.81E−01	0.34 ± 0.08	A	0.31
ENSSSCG00000024596	9	1	rs81420190	14.32–14.32	4.59E−02	1.00E+00	− 0.30 ± 0.08	C	0.24
ENSSSCG00000015129	9	4	rs81408352	24.50–28.89	6.62E−03	1.28E−01	− 0.27 ± 0.06	G	0.10
ENSSSCG00000015129	9	28	rs81318704	35.15–39.98	6.62E−03	1.39E−01	− 0.25 ± 0.06	A	0.12
ENSSSCG00000015129	9	10	rs81410722	45.90–47.65	2.64E−05	5.28E−05	− 0.25 ± 0.04	G	0.17
ENSSSCG00000015129	9	3	rs81226448	54.24–54.49	2.97E−02	7.72E−01	− 0.15 ± 0.03	C	0.28
ENSSSCG00000015129	9	1	rs81412811	65.10–65.10	4.49E−02	1.00E+00	− 0.19 ± 0.05	C	0.12
ENSSSCG00000017895	12	9	rs81436670	50.91–53.86	4.50E−07	1.36E−06	0.40 ± 0.06	G	0.33
ENSSSCG00000023829	14	1	rs80875259	31.27–31.27	1.24E−06	3.74E−02	− 0.36 ± 0.07	G	0.42
ENSSSCG00000014791	17	1	rs81466737	47.44–47.44	9.17E−07	2.76E−02	0.33 ± 0.07	A	0.22

SSC, *Sus scrofa* chromosome; N, number of SNPs significantly associated with the gene under study; SNP, corresponds to the most significant associated SNP; Region (Mb), region containing the SNPs significantly associated with the gene under study; *P*-value, nominal *P*-value of association between gene expression and SNP; *q*-value, *q*-value calculated with a false discovery rate approach; *B*, Bonferroni corrected *P*-values; δ, effect size of the marker and its standard error (SE); A_1_, minor allele; MAF, minor allele frequency

Finally, to verify whether the inclusion of these putative regulatory markers improves RFI prediction, the 269 eSNPs that affect genes associated to FE were added to the former SNP panel of 829 SNPs. The estimated proportion of RFI variance explained by these 1078 SNPs (Table 4) did not increase significantly (0.66 ± 0.06) compared to the variance explained by the 829 SNPs (0.61 ± 0.06) identified by AWM. The results from the subsequent cross-validation analysis showed that the correlation between observed and predicted RFI was slightly lower when the 269 eSNPs were added (dropped from 0.65 to 0.60; Table 4), which led us to conclude that there was no improvement in RFI prediction accuracy derived from including these putative regulatory markers in the predictive SNP panel.

## Discussion

In this study, we used a systems-based approach that combines SNP co-association and muscle gene expression analyses to identify candidate genes, biological pathways, and potential regulators of FE in pigs. An additional goal of our work was to develop a panel of SNPs that could predict FE phenotypes. The results obtained from both sources of information allowed us to recapitulate the known biological processes that affect FE (i.e. supported by current literature) and to identify novel biological pathways and candidate genes associated with this phenotype.

The AWM co-association network that was found to be associated with the nine evaluated phenotypes included 829 SNPs. The list of candidate loci that directly or indirectly affected FE included 879 genes, which are involved in a wide variety of biological processes. Among these, genes that displayed associations with a high proportion of the analysed traits were functionally related to either the nervous system function or development, as well as to the immune system. In agreement with this finding, and supporting their potential pleiotropic roles (since they were associated with several traits), it is worth mentioning that several of these genes were previously reported to be associated with FE traits (*SDK1*, *NGEF*, *CTNND2* and *JAK1*) [17, 48–50], fatness (*SDK1*, *JAK1*) [51, 52], growth (*CACHD1*, *NGEF*) [17, 53], or bone mineral density (*DNAJC6*) [54]. More interestingly, the AWM procedure allowed us to identify a group of TF genes that acted as hubs in the topology of the network, which suggests that they have a cooperative role in mediating a highly inter-connected regulatory cascade that seems pivotal for FE in pigs. This group of TF genes included *ESR1*, *SMARCA2*, *COPS5*, *GTF2H5*, *RUNX1*, *USP16*, *TCF7L2*, *MAML2* and *LHX4*, which have already been reported to be associated with FE in pigs and other livestock species [15, 18, 21, 26, 55–58].

The association between the skeletal muscle transcriptome and the traits that were most markedly correlated with FE was also explored, using different approaches. Results from DE between two groups of animals with extreme FE pointed out that less efficient animals have a higher protein turnover and increased energy expenditure compared to their highly efficient counterparts. Discriminant analysis showed that the gene expression profile of 200 genes (included in PC1) explained 24% of the global phenotypic variance in FE-related traits and allowed clear discrimination between animals with high and low RFI. This result allowed us to hypothesize that muscle gene expression data can have a predictive ability for classifying pigs into those having high or low FE, as observed by Piles et al. [59], who analysed liver and gut gene expression data using machine-learning algorithms. Based on results from the different transcriptomic analyses, we identified other potential regulators of FE among the genes that were functionally associated with FE. This group of potential regulator genes included *ESRRG*, *ZNF473*, *NFATC3*, *RXRG*, *PPARGC1A*, *NFKBIZ*, *TCEA1*, *CDCA7L*, *ZFP64*, *LZTFL1*, *RBL2*, and *CBFB*, which were previously reported to be related to FE [15, 16, 20, 21, 58, 60–63]. Among these genes, it is worth mentioning that *ESRRG* showed the largest differences in expression between animals with divergent FE and was among the top most relevant genes to explain the global phenotypic variance in FE-related traits in the multivariate analysis.

Combining the genes that were identified in both the genome and muscle transcriptome analyses resulted in an extensive list with more than 1000 candidate genes that were shown to have either a direct or an indirect effect on FE traits. According to their functional annotation, these genes are involved in a broad set of biological processes, indicating that the molecular mechanisms that control FE are highly interconnected and, in turn, are not only associated with energy metabolism, but also with immunity, behaviour, and the nervous system. Interestingly, the nervous system pathways included the melatonin signalling, glutamate receptor, and gustation pathways, which seem to play an important role in regulation of feed intake and FE [64–68]. Other pathways that were enriched in the list of candidate genes have also been previously reported as associated with FE traits [16, 28, 69], including aldosterone signalling in epithelial cells, ephrin receptor signalling, relaxin signalling, glycogen degradation, protein kinase A signalling, axonal guidance signalling, semaphorin signalling in neurons, and RhoGDI signalling pathways.

One of the main advantages of our methodological approach lies in the joint interpretation of results from both structural and functional genomic studies. Indeed, they corroborate the fundamental role of the nervous system in the regulation of FE and, specifically, of the pituitary gland jointly with the hypothalamus and thyroid axis. First, several genes that are functionally related to the function and development of the nervous system were included in the AWM network associated to FE traits, including *SDK1*, *NPTX1*, *NGEF*, and *CTNND2*. Moreover, *LIM homeobox protein 4* (*LHX4*), a TF gene involved in the control of the differentiation and development of the pituitary gland [70], was included in the set of potential regulators detected in the co-association network. In addition, in the muscle transcriptome analyses other relevant regulator genes were part of the list of DE genes, such as *RXRG*, which is expressed in pituitary cells. *RXRG*-deficient mice display high metabolic rates and resistance to weight gain when fed a high-fat diet [71], two features which may be explained by interference with the thyrotrope axis and/or from effects specific to skeletal muscle [71]. Other genes associated with regulation of FE through the hypothalamus and thyroid axis were also functionally associated with FE traits in our transcriptomic analyses, such as *core*-*binding factor beta* (*CBFB*) or *cells inhibitor zeta* (*NFKBIZ*).

Another relevant observation from the joint interpretation of the genomic and transcriptomic analyses is the key role of estrogen signalling in the regulation of FE in pigs based on several findings that support this observation. On the one hand, the *estrogen receptor 1* (*ESR1*) gene was among the AWM top regulators of the co-association network. On the other hand, the *ESRRG* gene, which encodes an estrogen-related receptor, showed a muscle expression pattern that was clearly associated with FE variance. These results are consistent with previous studies at the transcriptomic [15, 20] and proteomic [55] levels that reported a link of the *ESR1* and *ESRRG* genes with FE in pigs. Estrogens control many cellular processes and modulate growth and maintenance of the skeleton, as well as the cardiovascular and nervous systems [72, 73]. In addition, increased estrogen receptor (ERα and ERβ) signalling suppresses energy intake and increases energy expenditure [74]. Several studies have shed light on the interaction between the estrogen signalling pathway and the nervous system, and provide elements to understand the regulatory role of estrogens on FE-related traits. At the level of the central nervous system, the hypothalamus is responsible for controlling feed intake (appetite), energy metabolism, and body weight [74], and it has been reported that estrogens display a nucleus-specific action within the hypothalamus to modulate energy balance [75]. Noteworthy, the nuclear estrogen receptor *ESR1*, which is expressed on the pro-opiomelanocortin neurons, acts by suppressing feed intake and increasing energy expenditure [74] when it is activated by the estrogen hormone. Furthermore, cross-talk has been reported between estrogen and the regulation of thyroid hormone-releasing receptor [76, 77].

Finally, from a more practical perspective, our aim was to evaluate potential applications of our results in animal breeding. Improving FE is one of the most relevant objectives of the pig industry. Several investigations have proven the possibility of applying successful selection processes for FE [1–3], but selecting for FE is challenging because obtaining reliable measures of individual feed intake is difficult and costly. Thus, identifying accurate predictive markers of FE could be of capital relevance for genetic selection for FE. The 829 SNPs identified by the AWM approach explained a significant proportion (61%) of the RFI phenotypic variance, which suggests that they have a promising potential for obtaining a molecular-based prediction of FE records. This panel of SNPs offered the most favourable scenario regarding RFI prediction, based on a genomic prediction accuracy of about 0.65, which was higher than the prediction accuracies for FE reported in previous studies on pigs (0.40–0.53) [11, 12], which used larger population sizes and a larger number of SNPs. The estimated proportion of variance explained by our panel of selected SNPs was also greater than the estimated RFI heritability. Previously, a simulation study suggested that selecting causal SNPs in the panel of predictor markers could increase the estimated prediction accuracy above what is expected for a given heritability [78]. However, we cannot rule out the possibility of an upward bias in the estimated genomic heritability due to the fact that the entire dataset was used to both selecting the SNPs and performing the validation processes. In any case, this bias would not invalidate the relevant increase in the explained variance and prediction accuracy when compared with those obtained with the whole SNP dataset (31K). These results support the relevance of preselecting SNPs to improve the prediction accuracy and that there is little benefit in simply increasing SNP density, in agreement with previous studies [78, 79]. It is also worth noting that prediction accuracy when the whole SNP panel was used was worse than that achieved with only pedigree information, revealing the uselessness of the whole SNP panel when the training data is of small size.

Inclusion of eSNPs in the panel of markers did not improve the prediction accuracy of RFI, in spite of the slight increase in explained RFI variance. Among other reasons, this may be because of the modest size of the sample that was used for our mRNA expression analyses, i.e. 104 pigs, which limited the power of the study. In addition, most of the genes identified by the AWM procedure were related to nervous system functions, which may be poorly expressed in muscle. This agrees with the limited number of genes that were detected using the AWM approach and that were expressed in GM, i.e. 290 out of 879. The study of other relevant tissues, such as the pituitary gland and hypothalamus, may contribute to identifying candidate genes for FE and functional variants that are associated with their activity that could be useful to predict RFI phenotype. In any case, our results allow us to conclude that the molecular markers that were identified using the AWM approach present some predictive ability for FE, thus opening the possibility of applying more accurate selection processes to improve FE in pigs with a shorter generation interval, since predictions of FE could be obtained early in the life of the animal.

## Conclusions

An integrative approach that combines different sources of information has made it possible to identify candidate genes, pathways, and predictors that may be important in the determinism of FE in pigs. A list of genes that may have direct or indirect effects on FE was elaborated by combining outputs from genomic and muscle transcriptome analyses. These genes are involved in a broad set of biological processes that are mainly related to immunity, behaviour, and nervous system, along with energy metabolism. The set of TF genes that were identified either as regulators of the co-association network or as functionally related to FE traits are potentially responsible of the modulation of a highly inter-connected regulatory cascade that seems pivotal for FE in pigs. The list of these main regulator genes includes *ESR1*, *ESRRG*, *RXRG*, *PPARGC1A*, *SMARCA2*, *COPS5*, *GTF2H5*, *RUNX1*, *USP16*, *TCF7L2*, *MAML2, LHX4*, *ZNF473*, *NFATC3*, *NFKBIZ*, *TCEA1*, *CDCA7L*, *ZFP64*, *LZTFL1*, *RBL2*, and *CBFB*. Joint interpretation of results from both the structural and functional genomic studies indicated that the estrogen signalling pathway, the pituitary gland, the hypothalamus, and the thyroid axis play fundamental roles in the regulation of FE in pigs. The additive effects of a panel of 829 selected SNPs explained 61% of the phenotypic variance of RFI, whereas the prediction accuracy of RFI phenotype based on cross-validation was 0.65. These results offer a promising perspective about the usefulness of molecular approaches to predict FE and open the possibility of more accurate selection processes for improving FE in pigs with a shorter generation interval, since predictions of FE could be obtained early in the life of the animal.

## Supplementary information

**Additional file 1: Figure S1.** Representation of the system-based approach employed to identify candidate genes and predictors of feed efficiency. **Figure S2.** Classification error rates for each component in the sparse partial least squares discriminant analysis (sPLS-DA); the optimal number of variables to select in each component is indicated as a diamond. **Figure S3.** Classification performance obtained with the partial least squares discriminant analysis (sPLS-DA).

**Additional file 2: Table S1.** Annotation of the 829 SNPs that were selected in the association weight matrix (AWM) procedure.

**Additional file 3: Table S2.** Description of genes identified as differentially expressed between the high and low feed efficiency groups (HFE and LFE).

**Additional file 4: Table S3.** Description of the genes identified in the partial least squares discriminant analysis (sPLS-DA).

**Additional file 5: Table S4.** Description of the genes identified in the regularized canonical correlation analysis (rCCA).

**Additional file 6: Table S5.** Functional annotation of the identified candidate genes.

**Additional file 7: Table S6.** Description of the SNPs significantly associated with the gene expression according to the expression genome-wide association studies (eGWAS).

## Data Availability

Microarray data belonging to selected samples were deposited in the Gene Expression Omnibus (GEO) public repository, and are accessible through GEO Series Accession Number GSE115484. The phenotypic and genotypic datasets used during the current study are available from the corresponding author on reasonable request.
