# Supplementary material for: Integrating genome-wide co-association and gene expression to identify putative regulators and predictors of feed efficiency in pigs
Source: Genet Sel Evol. 2019 Sep 2;51:48. doi: 10.1186/s12711-019-0490-6 (PMC6721172; doi:10.1186/s12711-019-0490-6)
Supplement: Supplementary file 1 — Additional file 1: Figure S1. Representation of the system-based approach employed to identify candidate genes and predictors of feed efficiency. Figure S2. Classification error rates for each component in the sparse partial least squares discriminant analysis (sPLS-DA); the optimal number of variables to select in each component is indicated as a diamond. Figure S3. Classification performance obtained with the partial least squares discriminant analysis (sPLS-DA). [file 12711_2019_490_MOESM1_ESM.docx]

**Additional Figure S1.** Representation of the system-based approach employed to identify candidate genes and predictors of feed efficiency.


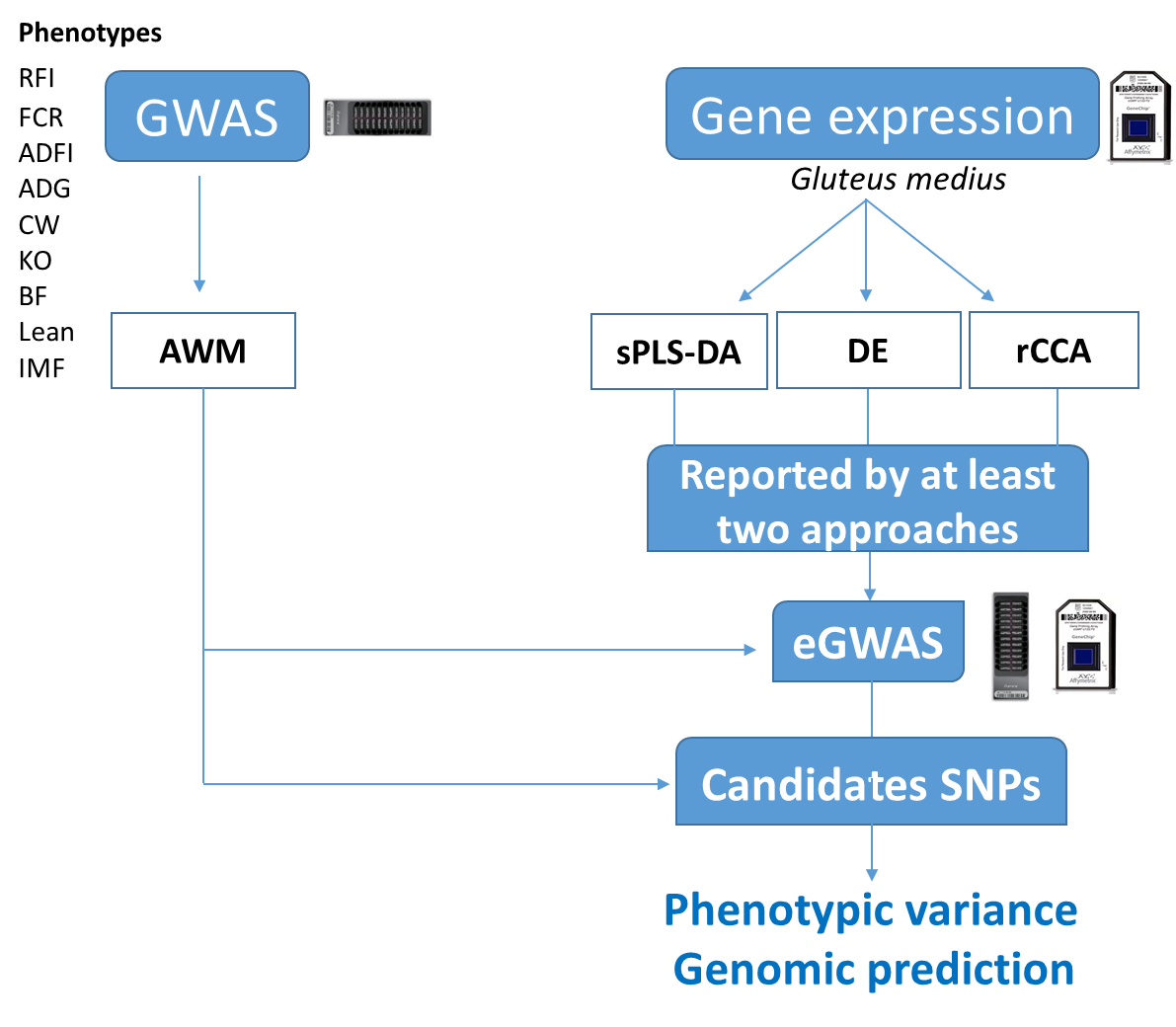


RFI: residual feed intake; FCR: feed conversion ratio; ADFI: average daily feed intake; ADG: average daily gain; BF: back fat thickness; CW: carcass weight; KO: killing out percentage; LEAN: lean percentage; IMF: intramuscular fat content

**Additional Figure S2.** Classification error rates for each component in the sparse Partial Least Squares Discriminant Analysis (sPLS-DA); where the optimal number of variables to select in each component is indicated as a diamond.


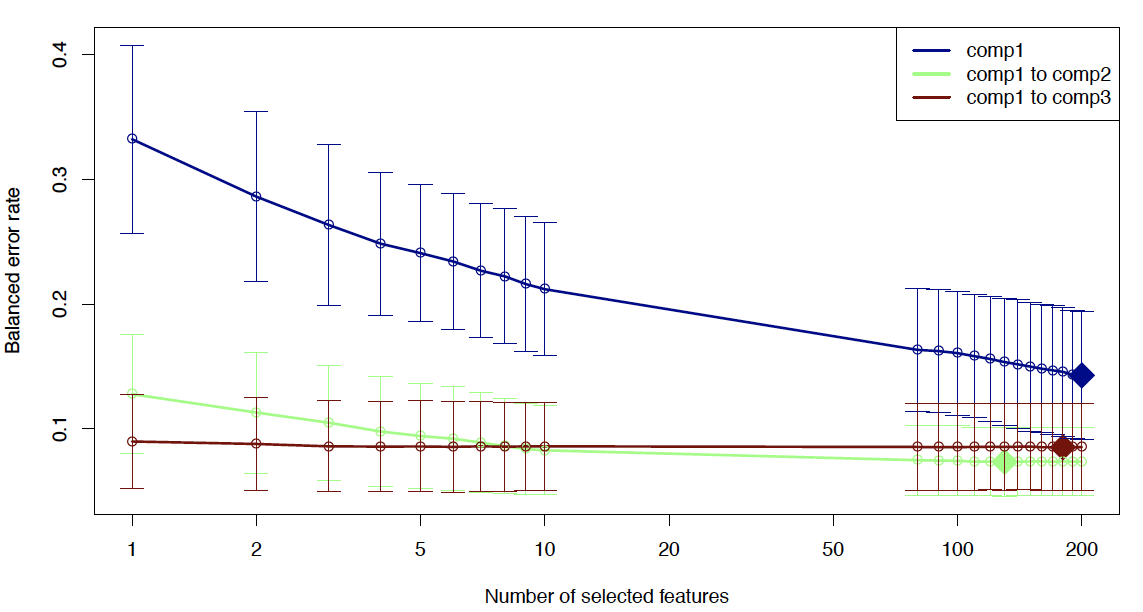


**Additional Figure S3.** Classification performance obtained with the Partial Least Squares Discriminant Analysis (sPLS-DA).

|  |  |
| --- | --- |
| 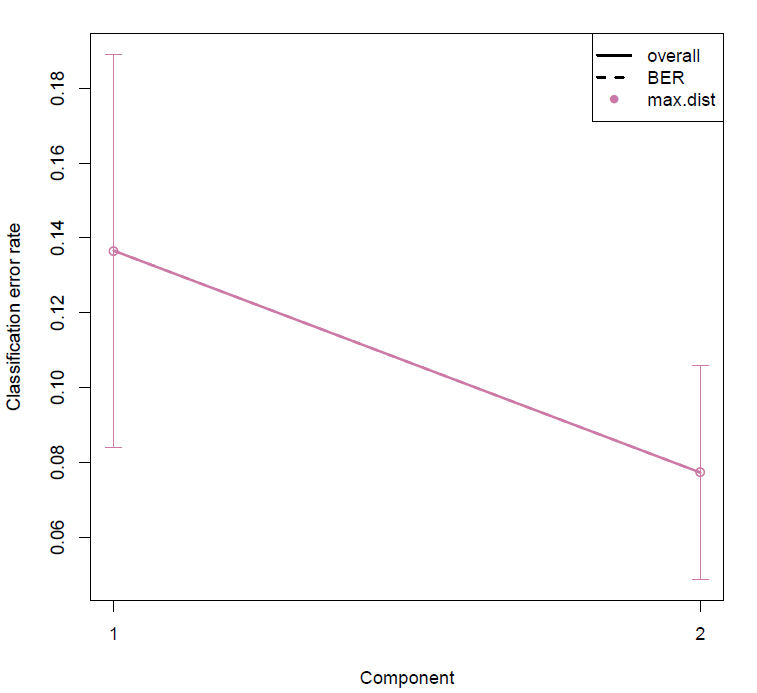 |  |
